# CTHNet: A CNN–Transformer Hybrid Network for Landslide Identification in Loess Plateau Regions Using High-Resolution Remote Sensing Images

**DOI:** 10.3390/s25010273

**Published:** 2025-01-06

**Authors:** Juan Li, Jin Zhang, Yongyong Fu

**Affiliations:** 1 College of Mining Engineering, Taiyuan University of Technology, Taiyuan 030024, China; lijuan0105@link.tyut.edu.cn; 2 Shanxi Institute of Surveying, Mapping and Geo-Information, Taiyuan 030001, China; 3 College of Resources and Environment, Shanxi University of Finance and Economics, Taiyuan 030006, China; yyong_fu@sxufe.edu.cn

**Keywords:** landslide detection, disaster extraction, deep learning, CNN, remote sensing

## Abstract

The Loess Plateau in northwest China features fragmented terrain and is prone to landslides. However, the complex environment of the Loess Plateau, combined with the inherent limitations of convolutional neural networks (CNNs), often results in false positives and missed detection for deep learning models based on CNNs when identifying landslides from high-resolution remote sensing images. To deal with this challenge, our research introduced a CNN–transformer hybrid network. Specifically, we first constructed a database consisting of 1500 loess landslides and non-landslide samples. Subsequently, we proposed a neural network architecture that employs a CNN–transformer hybrid as an encoder, with the ability to extract high-dimensional, local-scale features using CNNs and global-scale features using a multi-scale lightweight transformer module, thereby enabling the automatic identification of landslides. The results demonstrate that this model can effectively detect loess landslides in such complex environments. Compared to approaches based on CNNs or transformers, such as U-Net, HCNet and TransUNet, our proposed model achieved greater accuracy, with an improvement of at least 3.81% in the F1-score. This study contributes to the automatic and intelligent identification of landslide locations and ranges on the Loess Plateau, which has significant practicality in terms of landslide investigation, risk assessment, disaster management, and related fields.

## 1. Introduction

Landslides are a natural phenomenon in which soil or rock masses on a slope slide down along a certain weak level or zone under the influence of gravity [1,2]. They pose significant threats to human society and the natural environment by causing casualties and economic losses, and even triggering serious secondary disaster chains [3]. The Loess Plateau in northwest China is characterized by fragmented topography, ravines and gullies, and substantial fluctuation, making it susceptible to landslides [4,5]. According to statistical data, the Loess Plateau region accounts for one-third of the total number of landslides in the country, significantly impacting both local residents’ livelihoods and economic development [6]. Therefore, the development of efficient and accurate methods for landslide identification is of significance for the geological disaster emergency response, management, and risk assessment [7].

With the increasing applications of satellite remote sensing technology, landslide identification from images has largely replaced manual field investigations due to its rapid and large-scale investigative capabilities [8]. Traditional methods based on remote sensing images include visual interpretation, pixel-based methods, and object-based methods. Visual interpretation relies on expert knowledge and requires significant time and labor input, and it lacks prompt responsiveness [9]. Pixel-based and object-based methods enable automated interpretation, encompassing techniques such as threshold segmentation [10], region segmentation [11], edge detection [12], and clustering [13,14]. During this process, feature extraction techniques, including SIFT [15], SURF [16], and HOG [17], are commonly used to extract shallow characteristics like the spectrum, texture, and shape. However, due to the complex environment and various sizes of landslides, it is challenging to use shallow features extracted by expert knowledge and feature engineering to identify landslides with specific spectral, spatial, or temporal properties.

With the emergence of deep learning techniques, methods relying on feature engineering have gradually been replaced by convolutional neural network (CNN)-based methods [18]. These methods enable the acquisition of complex features and patterns from remote sensing images through multi-layer learning, enabling pixel-level segmentation for determining the landslide location and extent. Consequently, they are able to mitigate the limitations associated with the manual design to improve the accuracy and efficiency [19].

**Figure 1:**
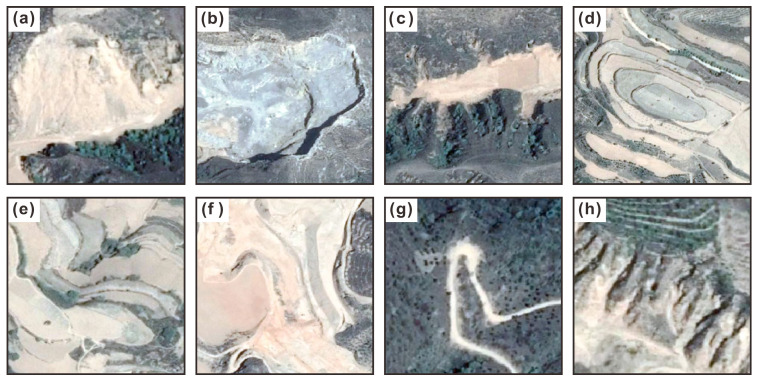
Surface features and landforms that are easily confused with loess landslides: (**a**) loess landslide, (**b**) artificially mined land, (**c**) gully bottom, (**d**) loess tableland, (**e**) cropland, (**f**) bare land, (**g**) road, and (**h**) gully.

However, current CNN-based models are facing significant challenges in accurately identifying loess landslides. The first challenge lies in the complex terrain and geomorphic environment of the Loess Plateau. The surface changes caused by loess landslides, such as the distinctive armchair shape, are high similar to various natural landforms and surface features in terms of the texture, color and shape dimensions, which can easily introduce confusion, resulting in misclassification and omission [20]. Specifically, as shown in Figure 1, the loess interfluve and gully landforms, such as the loess tableland (d) and gully (h), display linear or planar characteristics that are similar to the landslide boundaries in high-resolution remote sensing images (HRSIs). The sediment at the gully bottom (c) and bare land (f) share similar coloration with loess landslides. Cropland’s stepped structure (e) bears a resemblance to the step-like landforms formed by landslides. Excavation faces and deposits resulting from human activities, like mining (b), road construction, and building construction, possess shape and texture characteristics highly similar to landslide features in certain cases. All of these undoubtedly amplify the complexity of identification tasks.

The second challenge is the inherent limitations of CNNs. The critical global features of landslides, including the boundaries and location information, are easily captured by the visual system to distinguish landslides from similar natural landforms and surface features [21]. Despite the CNN model’s advantage in extracting local features, its ability to capture global context information is relatively limited, posing challenges in relation to distinguishing targets with similar local features in the complex environment from HRSIs [22,23]. Moreover, during the construction of feature maps, CNNs often use downsampling operations to enlarge the receptive field, which promotes feature abstraction but decreases the image resolution and crucial details, such as boundary and positioning information. Consequently, CNN models’ segmentation performance will be significantly diminished when faced with scenes characterized by consistent spectral properties or substantial differences in the target scale, especially for loess landslide areas [24].

To overcome the limitations of traditional CNN models, researchers have proposed various methods to expand the receptive field for capturing more multi-scale information, such as larger kernels, atrous convolution [25], and image-based or feature-based pyramids [26,27]. However, these approaches often result in increased parameters and loss of details, thereby reducing the effectiveness of features. Other approaches have introduced self-attention mechanisms, like Attention-UNet [28] and HCNet [18], to explore the dense, pixel-level context correlation. Nevertheless, they require significant memory consumption and computational costs to obtain global context information [18,19,22,24].

Compared with these CNN-based self-attention methods, transformer-based networks are able to provide a more effective approach, utilizing self-attention techniques to capture the long-distance dependencies between each patch [22,23]. The limitations of CNN-based methods can be overcome by incorporating the transformer model to enhance the acquisition of global information. Following this idea, we proposed a CNN–transformer hybrid model (CTHNet) using HRSIs for loess landslide identification. In order to carry out this study, we constructed a multi-channel landslide dataset form HRSIs through visual interpretation, data preprocessing, and data enhancement. We then evaluated and compared the performance of our proposed CTHNet with several CNN-based and transformer-based methods on this dataset. The main contributions are as follows:

(1) A database of 1500 landslide and non-landslide samples was constructed based on HRSIs, which were interpreted in Liulin County, a region within the Loess Plateau.
(2) A hybrid network framework integrating the strengths of the CNN and transformer was developed for loess landslide identification. An improved VGG-16 variant was used to construct the CNN module for detail mapping, and a multi-scale lightweight transformer (MLT) module was proposed to capture global scale information.
(3) The results and performance in terms of loess landslide identification were compared and analyzed with CNN-based and transformer-based methods.

## 2. Related Works

### 2.1. CNN-Based Methods for Image Segmentation

With the emergence of deep learning, traditional pixel-based and object-based methods that rely on feature engineering have been gradually replaced by CNNs [29]. Initially, a sliding window technique was used to train CNNs (e.g., AlexNet [30], VGG [31,32], GoogLeNet [33], ResNet [34]) to predict the category label for each pixel by considering its local region. However, this block-based method suffers from significant redundancy due to the repeated feature extraction in overlapping regions, as well as its inability to accurately capture location and context information. In contrast to early CNNs, the fully convolutional network (FCN) replaces the fully connected layer with a convolutional layer, enabling pixel-to-pixel mapping [35]. Subsequently, U-Net, based on an FCN and proposed by Ronneberger et al., introduces an encoder–decoder structure and skip connections, reducing the information loss during downsampling [36]. However, both the FCN and U-Net suffer from limited receptive fields and a lack of global context information, which ultimately hampers their segmentation accuracy [37]. To overcome these limitations, several methods have been proposed. Chen et al. proposed DeepLabV2, which incorporates atrous convolution layers and an ASPP module to effectively expand the receptive field [38]. Schlemper et al. proposed an attention-gated module that can be easily integrated into standard CNN models to improve the prediction accuracy by suppressing feature activation in irrelevant regions [28]. Additionally, Chong et al. proposed a hierarchical context network (HCNet) to differentially model homogeneous and heterogeneous pixels, which can not only hierarchically model global context information but also capture multigranularity representations to more robustly identify multi-scale objects [18].

The CNN-based methods have a strong ability to extract local features, but they are limited by their finite receptive fields and cannot capture global context information very well. When faced with the complex environment of HRSIs, they find it difficult to distinguish targets with similar local features.

### 2.2. Transformer-Based Methods for Image Segmentation

The transformer model, originally developed for natural language processing, introduces a self-attention mechanism that enables a focus on all the positions in the input sequence, effectively dealing with long-range dependencies. The vision transformer (ViT) achieves state-of-the-art ImageNet classification by directly applying the global self-attention transformer model to full-size images [39,40]. Subsequently, relevant studies have been conducted on the application of the transformer model in the computer vision field. Zheng et al. proposed SETR, which employs transformers as encoders to capture global context information in each layer and combines it with a simple decoder to construct a semantic segmentation network [41]. Liu et al. proposed a novel vision transformer model that incorporates a layered architecture and windowing self-attention mechanisms, enhancing the modeling flexibility across different scales through multi-scale windows [42]. He et al. introduced ST-UNet, which integrates the Swin transformer into the U-Net architecture to fuse local and global features and enhance the feature recognition of ground objects [43]. Chen et al. presented a hybrid transformer–CNN model based on the ViT, which combines the capability of CNNs in extracting local features with the advantage of transformers in capturing long-range dependencies [22].

The transformer-based methods excel at capturing global and long-range dependencies, but they are not as adept at capturing local features as CNNs. Consequently, some details are easily lost. In addition, the computational complexity of the self-attention mechanism increases quadratically when processing HRSIs, resulting in a higher computational cost.

### 2.3. Remote Sensing Image Segmentation for Landslides

With the continuous advancement of image-processing technology, numerous studies have been conducted on landslide mapping using CNN-based landslide identification technology. The research and improvement methods primarily focus on the following three aspects. (1) Additional auxiliary data from multi-modal sources are introduced to provide rich feature information. Liu et al. proposed the FFS Net method that fuses morphological features from HRSIs and topographic features from digital elevation model (DEM) data at a higher level, which improves the identification accuracy for old and new landslides [44]. Chen et al. developed a multi-input-channel U-Net landslide detection method that integrates SAR, optical, and terrain remote sensing data through an attention mechanism, not only improving the accuracy of landslide detection but also reducing false positive and false negative rates [45]. Xu et al. proposed FKGRNet, which integrates landslide knowledge extracted from a graph into the RetNet to enhance the accuracy of landslide recognition [46]. (2) Novel approaches for enhancing sample data. Liu et al. proposed a complex background enhancement method with multi-scale samples to improve the sample quality [47]. (3) Enhancement modules, attention mechanisms, or optimized loss functions are integrated to improve network structures. Xu Binzhi et al. introduced a pyramid structure based on ResNet and proposed a multi-scale deep attention mechanism to enhance the global feature perception [48].

Most methods used for landslide segmentation rely on trial-and-error architectures or simply utilize some general semantic segmentation models without considering the characteristics of HRSIs, which leads to suboptimal performance in handling these images. In response to the aforementioned challenges, researchers have begun experimenting with integrating CNNs and transformers into models for landslide detection. For instance, Wang et al. proposed a segmentation network designed for global information extraction and multi-scale feature fusion, which features a multi-branch mechanism integrating a transformer with a CNN to effectively distinguish landslides from other confusing objects [21]. Wu et al. proposed a semantic segmentation model named SCDUNet++, which combines a shallow CNN structure with a deeper Swin transformer structure to enhance the discrimination and extraction of landslide features [49]. Xiang et al. proposed TCNet, a network that employs a parallel-in-branch architecture, combining the transformer and CNN to capture both global context and low-level spatial details [50]. Nonetheless, the reliable identification of landslides using HRSIs data remains a major challenge.

## 3. Study Area

**Figure 2:**
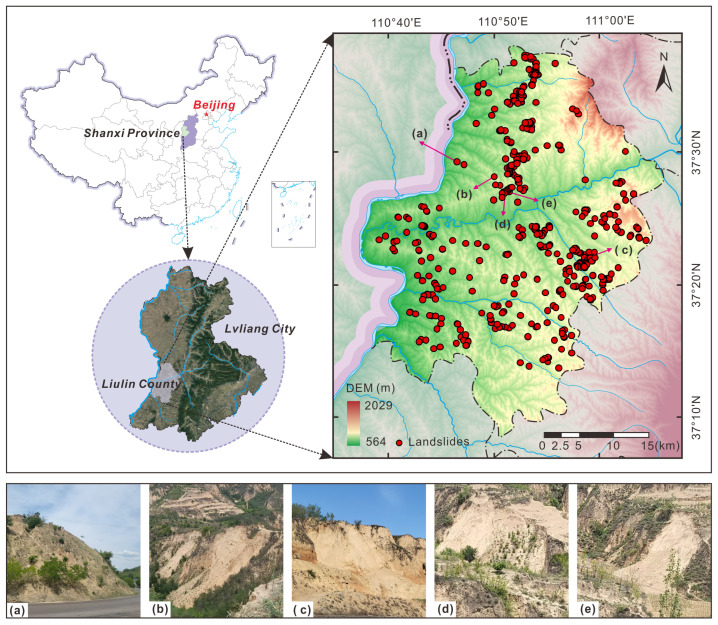
Geographical location and topographic variation within the study area, along with site investigation photos of landslides (**a**–**e**).

As depicted in Figure 2, the research area is located in Liulin County, Lvliang City, Shanxi Province, China, with a total area of 1287.29 km^2^. The region has a warm temperate continental monsoon climate characterized by four distinct seasons. The average annual temperature is 10.50 °C, with a range between −7.00 °C and 22.70 °C. The average annual precipitation from 2011 to 2020 was recorded at 538 mm.

Liulin County is situated within the hilly and gully region of the Loess Plateau in northwest China and has a diverse topography characterized by higher elevations in the northeast and lower elevations in the southwest. The surface is covered by Quaternary Loess, which has undergone gradual weathering, denudation, and water erosion over time, forming various complex geomorphic units. The main geomorphologic types comprise rolling loess hills (78.39%), with the remainder being middle–low mountains, valleys, and intermountain gullies. Geologically, Liulin County has a relatively simple structure, with the tectonic lines predominantly striking in a north–-south orientation. The primary structural features observed are faulting and folding. The lithology is primarily composed of mudstone, sandstone, silty clay, Ordovician limestone, and loose loess deposits. The region is rich in mineral resources, especially coal, which is widely distributed and easily exploitable.

Liulin County is dominated by loess landslides with clear boundary outlines and obvious shapes, as shown in Figure 2a–e. The complex natural geography and geological conditions provide favorable topographic and geomorphologic conditions for landslide geological disasters. Meanwhile, mining activities have aggravated the deterioration of the ecological geological environment, leading to frequent occurrences of natural disasters such as landslides [51].

## 4. Materials and Methods

**Figure 3:**
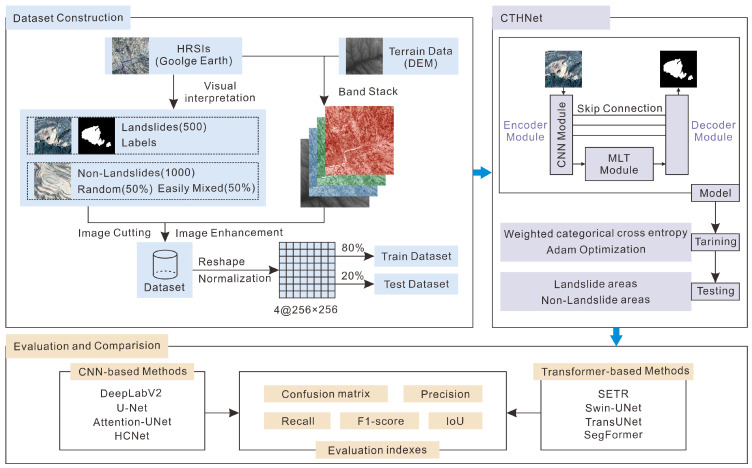
Flowchart of our study. ‘HRSIs’ represents high-resolution remote sensing images. ‘CTHNet’ represents our CNN–transformer hybrid network. ‘MLT’ represents the multi-scale lightweight transformer module.

As shown in Figure 3, our methodological workflow can be divided into three parts: dataset construction, CTHNet construction, and evaluation and comparison. Firstly, a landslide sample dataset was constructed from HRSIs through visual interpretation, data preprocessing, and data enhancement. This dataset was then divided into training and test sets for deep learning model training. Subsequently, a network model using a CNN–transformer hybrid as an encoder was constructed to extract landslide areas from our landslide sample dataset. Finally, the proposed method was evaluated and compared with several CNN-based and transformer-based methods, including U-Net [36], DeepLabV2 [39], Attention-UNet [28], HCNet [18], SETR [41], Swin-UNet [42], TransUNet [22], and SegFormer [52].

### 4.1. Data and Preprocessing

Due to the diversity of landslides and complexity of the surrounding environment, simply relying on HRSIs for identification may result in severe misclassification and omission [45]. Therefore, in this study, HRSIs and DEM data were fused in the channel to construct a landslide sample dataset for deep learning, as the formation of landslides is closely related to the terrain characteristics. As shown in Table 1, the HRSIs data were sourced from Google Earth images (https://earth.google.com/), and the terrain data were sourced from DEM data (https://search.asf.alaska.edu/) generated by the Advanced Land Observing Satellite (ALOS) phased array L-band synthetic aperture radar (PALSAR).

In this study, the landslide dataset was constructed based on historical landslide data with field verification. ArcMap 10.2 software was utilized for the identification and interpretation of landslides within the imagery. This process resulted in the creation of 500 landslide vectors, whose locations are depicted in Figure 1. These vectors were then converted into raster data to create binary imagery for use as labeled data. In this imagery, white pixels indicate landslide areas, while black pixels denote non-landslide areas. Cross-verification was performed on the interpretation results to control the quality of the labeled data. The non-landslide dataset consisted of 1000 samples, which were categorized into two types: (1) samples obtained through random sampling in areas not identified as danger-hidden points or historical landslides, and (2) easily confused samples. The two types were prepared at a ratio of 1:1 to maintain the sample randomness while ensuring an adequate number of easily confused samples.

Subsequently, ArcMap10.2’s model builder was used to perform a batch clipping based on selected sample areas and labels. The cropped dataset was divided into a training set and a test set in a ratio of 8:2, after ensuring its integrity. The training set was used for parameter and weigh training, and the test set was employed to evaluate its performance and determine the optimal parameters. Several data augmentation strategies, including rotation and symmetry, were applied to augment the training set, with a five-fold expansion to accelerate the network convergence speed and mitigate overfitting risks. All the samples were reshaped to dimensions of 256 × 256, followed by normalization of the image pixel intensity values within the range [0, 1].

### 4.2. Network Architecture

**Figure 4:**
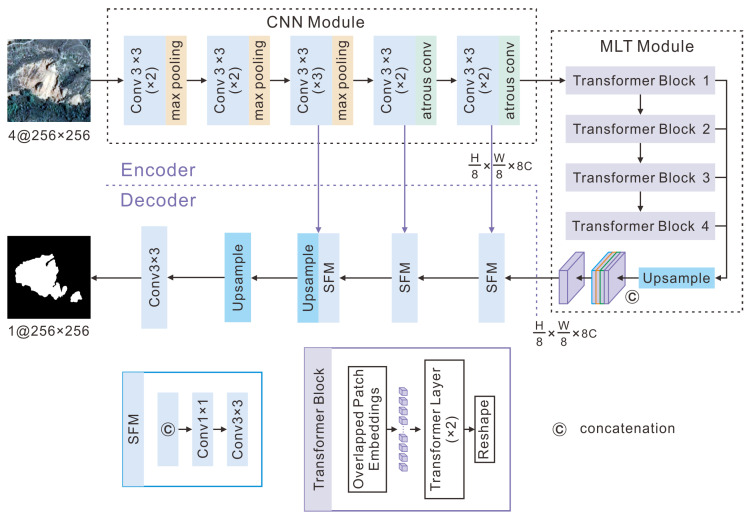
The overall network architecture of our proposed CNN–transformer hybrid network. ‘MLT’ represents the multi-scale lightweight transformer module. ‘SFM’ represents splicing and fusion.

As illustrated in Figure 4, our proposed model adopted an encoder–decoder architecture. The encoder employed a hybrid CNN–transformer design, and the decoder incorporated a series of cascaded upsampling layers to progressively restore images and ultimately obtain the segmentation results. Specifically, our model used a CNN module—a variant network of VGG-16—to encode local features from input images and extract high-dimensional feature maps. Subsequently, these feature maps were fed into an MLT module in which they were divided into fixed-size overlapping patches. Each patch’s pixel vectors were then flattened and fed into layered lightweight transformer layers. These transformer layers utilized self-attention mechanisms to learn global-scale features and capture the long-term dependencies between patches. Finally, the decoder was employed to upsample the self-attention features encoded by the MLT module, and it then fused these features with the corresponding level CNN feature maps in order to gradually recover local spatial information related to landslides. This process enhanced the details and enabled accurate landslide positioning. Detailed architectures of both the encoder and the decoder are provided in the following sections.

#### 4.2.1. CNN-Transformer Hybrid as Encoder

The encoder architecture of the CTHNet included a CNN module and an MLT module. The high-dimensional feature maps were extracted by the CNN module and were subsequently embedded into the MLT module. The following is a detailed introduction to these two modules.

(1) CNN Module

VGG-16 is a commonly used CNN model that constructs a deep network by stacking multiple blocks of convolutional and pooling layers [53]. However, due to its large number of parameters, it requires more time to train. Traditional pooling layers reduce the resolution of feature maps and the number of parameters, but at the expense of important spatial information. As shown in Figure 4, a variant network of VGG-16, used as a CNN module, deals with these issues. Fully connected layers were removed and the network consisted of 5 convolutional blocks, and each of which comprised a specific number of 3 × 3 convolution operations with a stride of 1. Specifically, each block contained 2, 2, 3, 2, and 2 convolution operations, respectively. Following each convolutional layer, batch normalization and the ReLu activation function were added to accelerate the network training. After each convolutional block, a 2 × 2 max-pooling layer was added to perform downsampling operations. Meanwhile, the number of feature channels was doubled during each downsampling process. To compensate for the reduced receptive field resulting from the omission of downsampling operations, the pooling layers in the 4th and 5th convolution blocks were replaced by atrous convolution with a dilation rate of 2. This improvement enhanced our model’s ability to capture complex landslide details and improved the localization accuracy without reducing the feature map resolution.

For a given input image $X\in R^{H\times W\times C}$, with a spatial resolution of H×W pixels and C channels, the image was downsampled 8 times throughout the VGG-16 network, resulting in a final high-dimensional feature maps of size $\frac{H}{8}\times \frac{W}{8}\times 512$. Let ${\{F}_{i}|i=1,2,3,4,5\}$ denote the feature maps obtained through the i-th convolution block, then there exists feature maps $F_{1},F_{2},F_{3},\;F_{4}\;and\;F_{5}$ with sizes $\frac{H}{2}\times \frac{W}{2}\times 64$, $\frac{H}{4}\times \frac{W}{4}\times 128$, $\frac{H}{8}\times \frac{W}{8}\times 256$, $\frac{H}{8}\times \frac{W}{8}\times 512$, and $\frac{H}{8}\times \frac{W}{8}\times 512$.

(2) Multi-Scale Lightweight Transformer Module

As shown in Figure 4, the MLT module consisted of four transformer blocks, each of which combined patch embedding and two transformer layers. The feature maps in each transformer block were upsampled to a resolution of $\frac{H}{8}\times \frac{W}{8}$, and then concatenated on the channel and fused by a 1 × 1 convolution layer to form the final multi-scale global feature maps of size $\frac{H}{8}\times \frac{W}{8}\times 512$. This design enabled the MLT module to generate multi-scale global features and improved the semantic segmentation performance.

**Figure 5:**
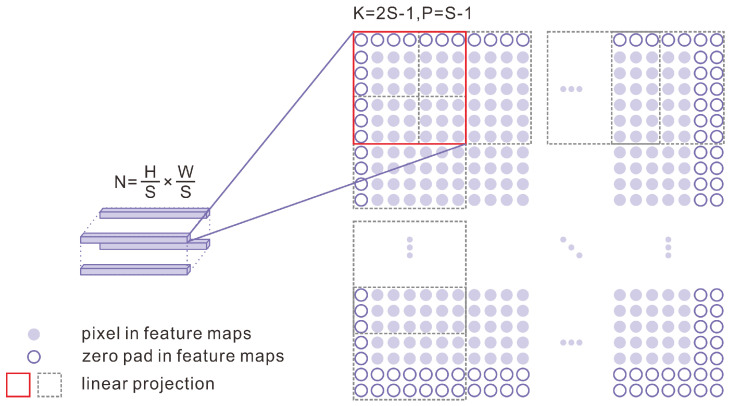
Overlapping patch embedding process.

Within conventional ViT models, images are treated as non-overlapping patches, resulting in the loss of local continuity; hence, an overlapping patch embedding technique was used on the CNN module’s feature maps, padding with zeros to maintain the resolution, as depicted in Figure 5 [54]. Specifically, for feature maps with dimensions H×W×C, we performed linear projection using a zero-fill convolution operation with stride S, kernel size 2S-1, and padding size S-1 to obtain N ($N=\frac{H}{S}\times \frac{W}{S}$) embedded patches.

**Figure 6:**
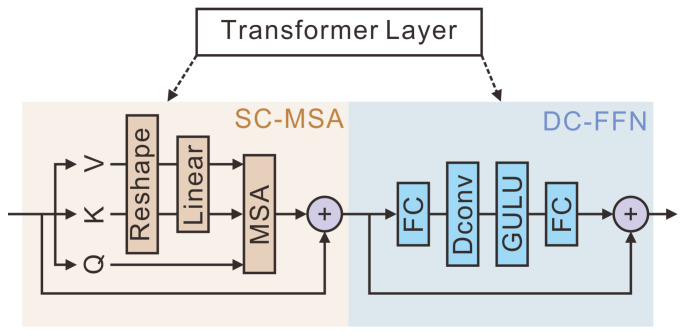
Structure of the transformer layer in our study. ‘SC-MSA’ represents the spatially condensed multi-head attention. ‘DC-FFN’ represents the depth-wise convolution feed-forward network. ‘FC’ represents a fully connected layer.

As shown in Figure 6, a transformer layer consisted of the spatial condensed multi-head attention (SC-MSA) and the depth-wise convolution feed-forward network (DC-FFN) [52]. To reduce the computational costs caused by attention mechanisms, SC-MSA employed a reduction process to decrease the number of key–value pairs before performing attention operations, with the reduction formula detailed as follows [24]:

(1) $$K_{new}=Linear(Reshape\left(K_{old},\frac{N}{R},D\cdot R\right),D)$$

(2) $$V_{new}=Linear(Reshape\left(V_{old},\frac{N}{R},D\cdot R\right),D)$$

where $K_{new}$ and $V_{new}$ are the key and value after reduction, $K_{old}$ and $V_{old}$ are the key and value before reduction, N denotes the number of key–values, D represents the length of a key–value, and R signifies the reduction rate. ${Reshape(K}_{old},\frac{N}{R},D\cdot R)$ is a linear operation that projects the feature onto a feature map of dimension D.

Therefore, attention scoring can be calculated using the following formula [55]:

(3) $${Attention(Q,K_{new},V}_{new})=Softmax\frac{\left(QK_{new}^{T}\right)}{\sqrt{D_{K}}}V_{new}$$

where Q, $K_{new}$ and $V_{new}$ are the query, key, and value, respectively. $D_{K}$ is the length of the $K_{new}$.

The conventional ViT model employs a fixed size for position coding. When HRSIs are input, the length of the sequence becomes longer, leading to the invalidation of both the position coding and weights [56]. Therefore, a 3 × 3 deep convolution with a fill size of 1 was introduced between the first fully connected layer (FC) and the GELU activation function in the DC-FFN module to dynamically generate conditional position coding [54]. This was able to generalize longer sequences, preserve the translation invariance required for image classification tasks, and enhance the classification accuracy. The SC-MSA and DC-FFN modules at layer L can be expressed as follows:

(4) $$z_{l}^{'}=\left(SC-MSA\right)\left(LN\left(z_{l-1}\right)\right)+z_{l-1}$$

(5) $$z_{l}=FC(GELU(DConv(FC(LN(z_{l}^{'})))))+z_{l}^{'}$$

where $z_{l}$ represents the output result of the L-layer transformer layer and $z_{l}^{'}$ represents the output result of the SC-MSA module. SC−MSA represents reduction and multi-head attention operator. LN represents the layer normalization operator, FC refers to the fully connected layer, DConv stands for the deep convolutional layer, and GELU denotes the GELU activation function.

#### 4.2.2. Decoder

The feature maps generated by the encoder are smaller than the original image. Directly upsampling them to the full resolution H × W inevitably results in the loss of low-level details. Therefore, a coarse-to-fine strategy that combines skip connections was adopted to aggregate multi-scale features from the encoders and upsampled features for the gradual recovery of landslide details. As depicted in Figure 4, multiple upsampling modules using bilinear interpolation and convolution modules were used to implement this strategy. Specifically, we initially concatenated the encoder’s features with the upsampled features of the same resolution through skip connections to create a richer feature representation. Subsequently, a combination of 1 × 1 and 3 × 3 convolutional layers was employed for feature fusion and channel reduction. This process of splicing and fusion was encapsulated within the SFM module. Finally, the resulting feature maps were upsampled by factors of 2× and 4× to match the original image size, followed by mapping these features to their respective categories using a 3 × 3 convolutional layers.

#### 4.2.3. Model Training

The experiment utilized the Python language and TensorFlow as the underlying framework. The training of the CTHNet model was executed on a workstation equipped with an Intel(R) Xeon(R) Gold 6242 CPU (Intel, Santa Clara, CA, USA) running at 3.10 GHz and an NVIDIA GeForce RTX 3090 GPU (NVIDIA, Santa Clara, CA, USA). The process was iterated 60 times, with a batch size of 60 and a learning rate of 0.0001, as shown in Table 2. The patch embedding used zero-fill convolution with kernel sizes of 7, 5, 3, and 3, strides of 4, 2, 2, and 1 in each transformer block. The number of heads employed in the multi-head attention mechanism within each transformer block was set as follows: 1, 1, 2, and 2, while the reduction rates for each block were set as: 8, 4, 2 and 1. The optimizer adopted adaptive moment estimation (Adam), which enabled adaptive adjustment of the learning rate for each parameter and enhanced the computational efficiency. To deal with the class imbalance during the training process, we employed the weighted cross-entropy loss function (WCE), which incorporated weight factors for individual categories into the calculation of cross-entropy loss. The formula is presented as follows [57]:

(6) $$L_{WCE}=-\frac{1}{N}\sum_{i=1}^{N}\sum_{j=1}^{K}W_{j}*y_{i,j}*log(p_{i,j})$$

where N represents the total number of samples, $W_{i}$ denotes the weight factor of the i-th sample used to adjust the contribution degree of different samples, K represents the total number of categories, $y_{i,j}$ signifies the true label that belongs to class j for sample i, and $p_{i,j}$ indicates the probability value predicted by the model for sample i belonging to class j.

The current study dealt with a binary classification problem with K set to 2. The corresponding label y was 0 or 1. The variable $p_{i}$ represents the predicted probability of belonging to the label y. Consequently, the loss function can be simplified as presented in the following equation:

(7) $$L_{WCE}=-\frac{1}{N}\sum_{i=1}^{N}\left[W_{1}*y_{j}*\log\left(p_{i}\right)+W_{0}*{(1-y}_{i})*log(1-p_{i})\right]$$

### 4.3. Accuracy Evaluation

The results of the pixel segmentation are divided into four categories: true positive (TP), true negative (TN), false positive (FP), and false negative (FN), as shown in the confusion matrix in Table 3. To quantitatively evaluate the performance of CTHNet in landslide identification, four commonly used semantic segmentation metrics, in terms of the precision, recall, F1-score, and IoU, were computed based on the confusion matrix and used for accuracy evaluation.

Precision represents the proportion of correctly predicted positive samples to the total number of positive predictions, serving as a measure of the model’s accuracy in predicting positive samples. Recall refers to the ratio of correctly predicted positive classes to all the actual positive classes. The F1-score provides a comprehensive performance evaluation for imbalanced datasets by considering the balance between precision and recall. The IoU is an index for evaluating the overall segmentation accuracy by quantifying the overlap between predicted and actual bounding boxes at the pixel level across different categories. The calculation formulas are as follows:

(8) $$Precision=\frac{TP}{TP+FP}$$

(9) $$Recall=\frac{TP}{TP+FN}$$

(10) $$F1=\frac{2\times Precision\times Recall}{Precision+Recall}$$

(11) $$IoU=\frac{TP}{TP+FN+FP}$$

## 5. Results

In order to assess the performance of the CNN–transformer hybrid model in loess landslide identification, we conducted a comprehensive evaluation through comparative experiments based on the landslide dataset concerning Liulin County. In these comparative experiments, we considered several CNN-based and transformer based approaches. The CNN-based methods included U-Net, DeepLabV2, Attention-UNet, and HCNet, while the transformer-based methods comprised SETR, Swin-UNet, TransUNet, and SegFormer. In order to conduct a comprehensive and quantitative evaluation, we calculated various evaluation metrics based on the confusion matrix. The evaluation results for the CNN-based and transformer-based methods are presented in Table 4 and Table 5, respectively.

As shown in Table 4, there was a small gap in the precision index among the CNN-based methods, with our proposed method achieving the optimal result and leading by at least 4.55%. When considering recall, our proposed method had significant advantages over the other methods by 2.11%, 6.49%, 9.58%, and 16.97%, respectively. Furthermore, our proposed model outperformed other methods in terms of the F1-score index for the balanced accuracy rate and recall rate, as well as the comprehensive evaluation index IoU, achieving superior values that were at least 3.81% and 4.56% higher than those obtained by other methods.

When comparing the evaluation metrics based on transformer methods, our proposed model showed significant improvements in precision and recall, and it demonstrated evident advantages. Moreover, all the metrics surpassed those of the comparison network by at least 4.55%, 3.88%, 4.2%, and 5.01%.

To further demonstrate the superiority of the proposed model in landslide identification, we compared the results obtained from various methods with real ground images and labels (the red lines in the image represent the outlines of the landslide areas).

**Figure 7:**
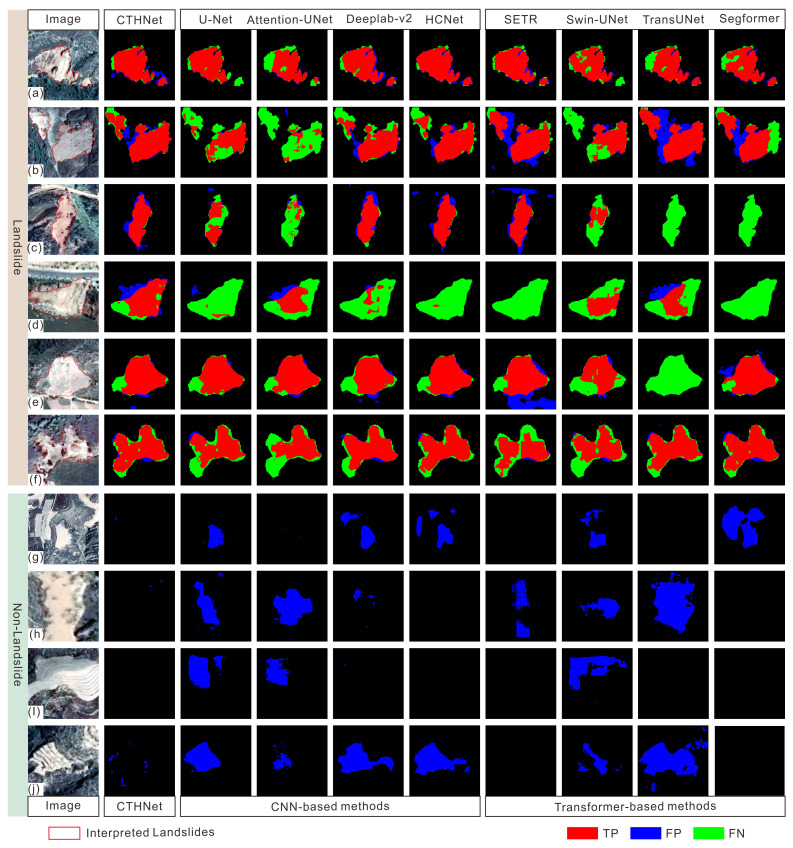
Segmentation results of different models in complex landslide environments: (**a**–**f**) segmentation results of landslide samples, (**g**–**j**) segmentation results of non-landslide samples, and red lines in images represent interpreted landslides. The red-covered part represents the true positive, the blue-covered part represents the false positive, and the green-covered part represents the false negative.

As depicted in Figure 7, the landslide range and boundary predicted by the CTHNet model are closely align with the actual ground conditions, which integrate local features and global context information for a more accurate representation. In contrast, both CNN-based and transformer-based methods exhibit varying degrees of issues, such as missed detection, false detection, and inaccurate identification of the landslide scope and boundaries, due to inadequate global context information and detailed specifics.

## 6. Discussion

### 6.1. Comparative Result Analysis

**Figure 8:**
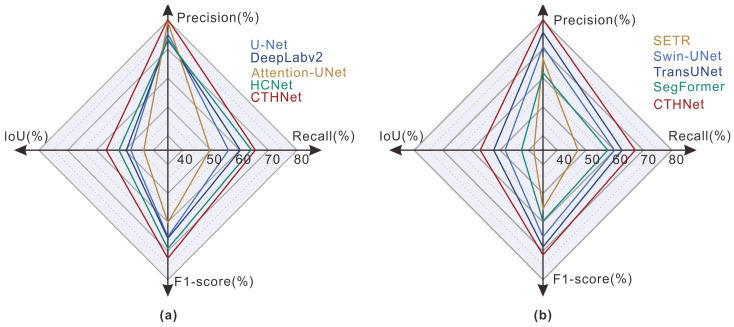
Radar chart depicting the evaluation indices: (**a**) the result of CNN-based methods; and (**b**) the result of transformer-based methods.

The outcomes of each quantitative evaluation metric have been visually represented in Figure 8 using a radar map, which is a method for displaying the distribution of data across multiple variables. It is evident that our proposed model demonstrates superior overall performance. From the perspective of quantitative evaluation metrics, the proposed model exhibits high scores in terms of the precision, recall, F1-score, and IoU, indicating its ability to accurately identify a greater number of landslide samples and successfully achieve a balance between the accuracy and recall rates to overcome both false positives and missed detection.

In terms of the methodology, the U-Net approach combines an encoder–decoder structure and skip connections to better acquire contextual and positional information. The Attention-UNet method achieved a remarkable precision value of 79.16% in landslide identification by integrating an attention gate model into U-Net’s skip connections to suppress feature activation in irrelevant regions. It is worth noting that both methods exhibit satisfactory performance in local feature extraction, and skip connections help reduce information loss during the downsampling operation. However, this architectural design primarily focuses on feature fusion and restoration rather than extracting global context information. In the case of Figure 7b–d, there were some missed detections to some extent. The DeepLabV2 method introduces an ASPP module to capture multi-scale information through multiple dilated convolution layers of varying sampling rates in parallel to enhance the detection capability for small landslides. However, the details of the landslide boundaries are not adequately preserved due to the lack of detailed information at different levels, as illustrated in Figure 7a. Furthermore, valuable information may be lost during the fusion of feature maps at different scales, so that the global background cannot be inadequately integrated, leading to false detections. This issue is reflected in Figure 7g,j, where terraces are inaccurately identified as landslides. The HCNet introduces an attention mechanism to partially compensate for the limitations of convolutional operations; however, its effectiveness is still constrained by the implementation method and network structure design. As depicted in Figure 7c, certain road areas are classified as landslides.

For the transformer-based methods, SETR employs a pure transformer structure as the encoder to replace the CNN encoder, and low-resolution feature maps with inadequate detailed positioning information are not recovered by direct upsampling to full resolution. SegFormer employs multi-layer perceptrons for information aggregation across different layers, while Swin-UNet integrates feature information from various scales through skip connections, thereby supplementing detailed information to a certain extent in comparison with SETR. TransUNet integrates the CNN encoder’s structural design to enhance the extraction of precise landslide boundaries. Compared with these transformer-based methods, our proposed model introduced an MLT module placed behind a variant VGG-16 as an encoder. This design is superior for obtaining feature maps with smaller spatial resolution that contain richer deep semantic information and a larger receptive field. Additionally, the lightweight design of the MLT module is beneficial for reducing computational costs. The results show excellent performance in suppressing the false positives in the segmentation results.

In summary, the proposed model shows better comprehensive performance in identifying complex loess landslides compared to various CNN- and transformer-based methods. It effectively captures both global and local landslide characteristics, thereby enhancing the ability to distinguish between landslide and non-landslide features. Notably, it provides segmentation results that closely align with the ground truth data, with lower false rates and missed detections.

### 6.2. Ablation Analysis

**Figure 9:**
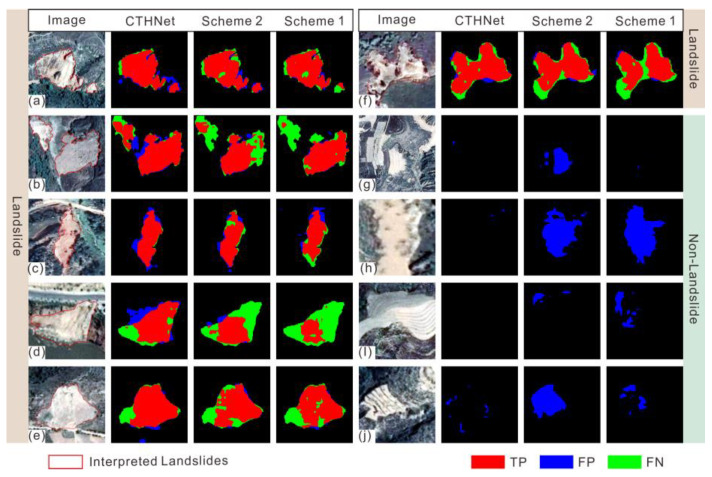
Comparison of the segmentation results from the ablation experiments: (**a**–**f**) segmentation results of landslide samples, (**g**–**j**) segmentation results of non-landslide samples, and red lines in the images represent interpreted landslides. The red-covered part represents the true positive, the blue-covered part represents the false positive, and the green-covered part represents the false negative.

To further investigate the impact of different structures on the segmentation results, we conducted ablation experiments in this study. Specifically, we first excluded the MLT module and skip connections from our model, then we gradually reintroduced them for comparison. Three distinct groups of schemes were designed, and the quantitative evaluation metrics and segmentation results for each scheme are presented in Table 6 and Figure 9.

As illustrated in Table 6, Scheme 2 shows minimal changes in precision compared to Scheme 1. However, it is worth noting that the recall value increases notably by 2.21%. This suggests that the introduction of skip connections contributes to the improved identification of landslides. The introduction of the MLT module in Scheme 3 results in a notable enhancement of both the precision and recall, with increases of 5.5% and 2.7%, respectively, compared to Scheme 2. Additionally, the model achieves high values for comprehensive metrics such as the F1-score and IoU. Combined with the results depicted in Figure 9, we can see that the MLT module plays a key role in reducing misjudgments of landslides and enhances the overall performance.

## 7. Conclusions

In this study, a database of 1500 landslide and non-landslide samples was constructed based on HRSIs in Liulin County for landslide identification from complex loess backgrounds. To deal with the challenges of identifying landslides in the complex environments of the Loess Plateau using deep learning models, we proposed the CTHNet, which focused on three main aspects. (1) The structure of the VGG-16 variant was optimized in the CNN module by discarding part of the downsampling operation and introducing atrous convolution to more effectively extract context information in HRSIs. (2) The MLT module was placed behind the CNN module and was used to capture remote dependencies and global context information related to loess landslides. (3) Through skip connections, the shallow fine features in the encoder were used to gradually restore some details and multi-scale features of loess landslides. The experimental results further validated the effectiveness and advantages of our method, achieving an F1 score of 72.81%. Compared to other CNN- or transformer-based methods, our method can better focus on landslide characteristics, suppress unnecessary background noise, and enhance the accuracy in landslide identification in complex background images.

The developed model can quickly generate a landslide inventory map of the target area after acquiring the data and holds promise for applications in landslide investigation, risk assessment, disaster management, and related fields. Future studies may focus on integrating InSAR data to enhance the identification of deforming landslides.

## Figures and Tables

**Table 1:** The data used in our study.

Data Type	Data Sources
VHSR Image Data	Google Earth
Terrain Data (DEM)	ALOS

**Table 2:** Relevant parameters for the model training.

Relevant Parameter	Parameter Value
Epoch	60
Batch size	8
Learning rate	0.0001
Zero-fill convolution for patch embedded	Kernel sizes = [7,5,3,3] Strides = [4,2,2,1] C = 256
Head number of MSA	[1,1,2,2]
Reduction rate	[8,4,2,1]
Optimizer	Adam
Loss function	WCE

**Table 3:** Confusion matrix.

Confusion Matrix		Truth Value	
		Positive	Negative
Predictive Value	Positive	TP	FP (Type II)
	Negative	FN (Type I)	TN

**Table 4:** Quantitative comparison of different CNN-based models. The best results in each index are shown in bold.

Methods	Evaluation Metrics (%)			
	Precision	Recall	F1-Score	IoU
U-Net	76.26	56.92	65.19	48.35
DeepLabV2	74.56	60.01	66.50	49.81
Attention-UNet	79.16	49.53	60.93	43.82
HCNet	74.31	64.39	69.00	52.67
CTHNet	**80.42**	**66.50**	**72.81**	**57.23**

**Table 5:** Quantitative comparison of different transformer-based models. The best results in each index are shown in bold.

Methods	Evaluation Metrics (%)			
	Precision	Recall	F1-Score	IoU
SETR	66.68	47.17	55.25	38.17
Swin-UNet	70.94	60.10	65.07	48.22
TransUNet	75.87	62.62	68.61	52.22
SegFormer	61.94	57.58	59.68	42.53
CTHNet	**80.42**	**66.50**	**72.81**	**57.23**

**Table 6:** Quantitative comparison of the ablation experiments. Scheme 1: CTHNet without skip connection structure and MLT module. Scheme 2: Scheme 1 with the addition of a skip connection structure. Scheme 3: Our proposed model. The best results in each index are shown in bold.

Methods	Evaluation Index (%)			
	Precision	Recall	F1-Score	IoU
Scheme 1	74.96	61.59	67.62	51.08
Scheme 2	74.92	63.80	68.91	52.57
Scheme 3	**80.42**	**66.50**	**72.81**	**57.23**

## Data Availability

The data that support the findings of this study are available from the corresponding author upon reasonable request.

## Abbreviations

Adam	Adaptive Moment Estimation
ALOS	Advanced Land Observing Satellite
CNN	Convolutional Neural Network
CTHNet	CNN–Transformer Hybrid Network
DC-FFN	Depth-Wise Convolution Feed-Forward Network
DEM	Digital Elevation Model
FC	Fully Connected Layer
FCN	Fully Convolutional Network
FN	False Negative
FP	False Positive
HCNet	Hierarchical Context Network
HRSI	High-Resolution Remote Sensing Image
MLT	Multi-Scale Lightweight Transformer
SC-MSA	Spatial Condensed Multi-Head Attention
TN	True Negative
TP	True Positive
ViT	Vision Transformer
WCE	Weighted Cross-Entropy Loss Function
